# Biochemical Oxygen Demand Prediction Based on Three-Dimensional Fluorescence Spectroscopy and Machine Learning

**DOI:** 10.3390/s25030711

**Published:** 2025-01-24

**Authors:** Xu Zhang, Yihao Zhang, Xuanyi Yang, Zhiyun Wang, Xianhua Liu

**Affiliations:** School of Environmental Science and Engineering, Tianjin University, Tianjin 300354, China; zhangxu_2022@tju.edu.cn (X.Z.); zhangyihao_@tju.edu.cn (Y.Z.);

**Keywords:** BOD, three-dimensional fluorescence, PARAFAC, random forest model

## Abstract

Biochemical oxygen demand (BOD) is an important indicator of the degree of organic pollution in water bodies. Traditional methods for BOD_5_ determination, although widely used, are complicated and dependent on accurate chemical measurements of dissolved oxygen. The aim of this study was to propose a facile method for predicting biochemical oxygen demand by fluorescence signals using three-dimensional fluorescence spectroscopy and parallel factor analysis in combination with a machine learning algorithm. The water samples were incubated for five days using the national standard method, during which the dissolved oxygen contents and three-dimensional fluorescence spectroscopy data were measured at eight-hour intervals. The maximum fluorescence intensity of three fluorescence components was decomposed and extracted by parallel factor analysis. The relationship between the maximum fluorescence of the three fluorescence components and the BOD_5_ values was established using a random forest model. The results showed that there was a good correlation between the fluorescence components and BOD values. The BOD_5_ values were effectively predicted by the random forest model with a high goodness of fit (R^2^ = 0.878) and low mean square error (MSE = 0.28). Although this method did not shorten the incubation time, successful BOD_5_ prediction was realized by the non-contact measurement of fluorescence signals. This avoids the complicated operation of DO determination, improves detection efficiency, and provides a convenient solution for analyzing large quantities of water samples and monitoring facile water quality.

## 1. Introduction

The pollution control of water resources is an increasingly pressing challenge, driven by the intensification of human activities and the ongoing deterioration of ecosystems [1,2,3]. A number of factors, such as the discharge of industrial wastewater, the use of chemical fertilizers and pesticides in agricultural activities, and the indiscriminate discharge of urban sewage, are exacerbating the pollution of water bodies. This pollution not only threatens the freshwater resources on which mankind depends, but also has a huge impact on the stability of aquatic ecosystems. Regular water quality testing is essential for effective river management and timely pollution control. Water quality monitoring can help managers to understand the pollution status of water bodies in a timely manner, so as to provide a reliable basis for the development of scientific and reasonable management measures. Various chemical parameters, such as total organic carbon (TOC) [4], chemical oxygen demand (COD) [5], and biochemical oxygen demand (BOD) [6], are commonly used to evaluate the severity of water pollution. Among these, BOD is a key indicator for assessing the concentration of organic pollutants in a water body. Its value directly reflects the level of organic pollution in water or wastewater environments [7]. Higher BOD values indicate elevated levels of organic pollutants in water, requiring a greater consumption of dissolved oxygen (DO) to decompose these substances. This increased oxygen demand threatens the health and balance of aquatic ecosystems [8].

Several BOD detection methods based on different principles have been proposed, including microbial fuel cells [9,10], electrochemical processes [11], and optical [12] and biosensor methods [6]. Each of these methods offers distinct advantages and disadvantages (Table 1). For instance, microbial fuel cells provide rapid detection but are complex to maintain and lack sensitivity, while biosensors are compact and highly sensitive but have a limited operational lifespan. Electrochemical methods have also shown some potential by virtue of their efficiency and convenience, but they may be subject to interference in complex water samples, resulting in reduced accuracy. Optical methods have advantages in contactless detection, but they are costly and prone to interference in high-turbidity samples. In comparison, the national standard method is internationally recognized for its authority and comparability. However, despite its global acceptance, the traditional standard BOD detection method has certain limitations. Standard methods require a high level of operator skills and are prone to instability and the poor reproducibility of test results due to improper operation or minor errors in the handling process. These problems are especially likely to be exposed in large-volume sample testing. Therefore, how to improve the ease of operation and stability of results while maintaining the authority and accuracy of traditional methods has become one of the important topics in current research.

Three-dimensional (3D) fluorescence spectroscopy has gained attention for its ability to identify fluorescent components in complex mixtures. When combined with parallel factor analysis (PARAFAC), it can decompose these components to reveal the fluorescence characteristics of individual substances [13,14,15,16]. Water quality can be facilely estimated by analyzing the fluorescence information of organic matter in water samples. Meanwhile, the rapid advancement of machine learning has led to the continuous development of soft measurement models for BOD estimation. For example, Ching et al. [17] introduced a novel soft sensor for predicting BOD_5_ in wastewater using the gradient boosting (XGBoost) machine learning technique. The sensor was tested on influent and effluent BOD_5_ data from two different wastewater treatment plants, with modeling results demonstrating that XGBoost outperforms traditional soft sensors, particularly in detecting extreme values. Similarly, Pattnaik et al. [18] proposed a machine learning-based soft sensor model for estimating BOD, further highlighting the potential of these approaches. The experimental setup used test readings from 100 water samples to evaluate the performance of this technique with the statistical metrics of correlation coefficient (=0.9273), mean absolute error (=0.082), and root mean square error (=0.1994). It is expected that combining PARAFAC with machine learning (ML) for BOD_5_ prediction can offer promising new possibilities for enhancing the efficiency of BOD detection.

In this study, PARAFAC and ML methods are utilized to develop a BOD prediction model for domestic wastewater in a northern urban river. The 3D fluorescence spectroscopy data of the water samples were analyzed using PARAFAC, and the BOD_5_ test was performed using the national standard method. A random forest regression model was used to establish the relationship between spectral components and BOD values. Random forests are widely used in environmental studies due to their ability to effectively handle non-linear relationships and interactions between variables. Their robustness to noise and capacity to manage complex datasets make them a reliable choice for various applications, including water quality monitoring. Although the method developed in this study does not reduce detection time, it simplifies the experimental workflow and is easier to use for routine and large-scale water quality assessments. The findings of this study may provide new solution ideas for more efficient and automated BOD monitoring techniques.

## 2. Materials and Methods

### 2.1. Research Area and Sample Collection

**Figure 1:**
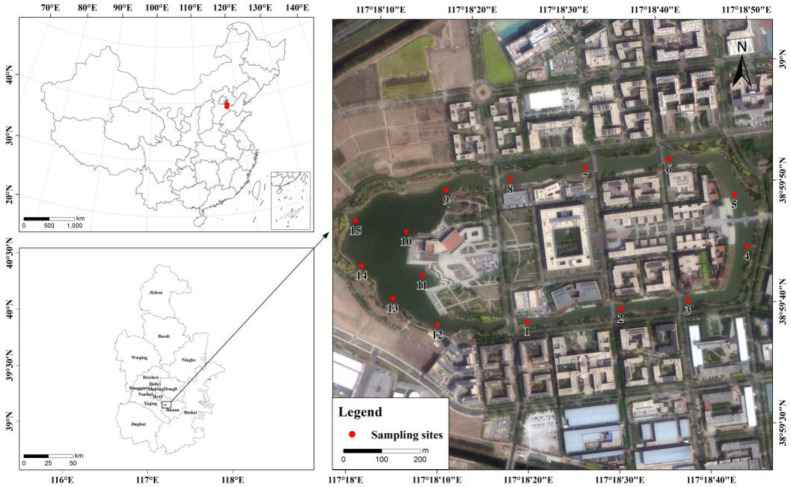
River study area and distribution of sampling sites.

Samples were collected from Qingnian Lake and the Neihuan River in Jinnan District, Tianjin, China. Fifteen sampling sites (S1–S15) were selected (Figure 1), encompassing nearly the entire water area. Sampling was conducted in 2024, during which water samples were collected in clean stainless-steel drums at designated locations to avoid sample contamination. At each site, 2 L of water was collected at a depth of approximately 20 cm below the water surface to ensure that the water samples were environmentally representative. It was then filtered through a 1.6 μm membrane to remove large amounts of suspended solids that could interfere with subsequent experiments. The filtered samples were transferred to clean brown glass bottles and stored at 4 °C until laboratory analysis. In the laboratory, the water samples were distributed into sixteen 100 mL brown glass bottles. Dissolved oxygen levels and 3D fluorescence spectra were measured at 8-h intervals, starting from the time-zero point of the experimental incubation. All water samples were collected at once and incubated at different times. DO measurements were made using a dissolved oxygen meter, and three-dimensional fluorescence spectra were determined with a fluorescence spectrophotometer. All brown glass bottles were kept sealed during the experiment to avoid outside air interfering with the oxygen concentration.

### 2.2. BOD Measurement Method

Considering the actual situation of river water quality, the biochemical oxygen demand at specific time intervals was determined by the non-dilution method. Under the specified aerobic conditions, we put a quantitative sample culture solution to be tested into a culture bottle and incubated it at 20 ± 1 °C. We determined the dissolved oxygen before incubation and the dissolved oxygen after sample incubation every eight hours. The biochemical oxygen demand that gives the difference between the two is the BOD value. The formula for calculating BOD_t_ is shown in Equation (1):

(1) $$BOD_{t}=\rho_{0}-\rho_{t}$$

where ${BOD}_{t}$ is the biochemical oxygen demand for a special time interval, mg/L; t is the incubation time, h; $\rho_{0}$ is the mass concentration of the pre-incubation dissolved oxygen (DO) of the water samples, mg/L; and $\rho_{t}$ is the mass concentration of the dissolved oxygen (DO) of the water samples after t hours incubation, mg/L.

### 2.3. Three-Dimensional Fluorescence Spectroscopy Measurements

Three-dimensional fluorescence spectra of water samples were determined using an F97XP fluorescence spectrophotometer. The instrumental setup parameters of the fluorescence spectrophotometer were as follows: excitation wavelengths from 200 nm to 500 nm, emission wavelengths from 250 nm to 550 nm, excitation and emission wavelength intervals of 5 nm, a scanning speed of 15,000 nm min^−1^, excitation bandwidths of 10 nm, emission bandwidths of 10 nm, slit widths of 5 nm, and a gain (PMT) of 900 V. Daily measurements of Milli-Q water were required as a blank control.

### 2.4. PARAFAC Modeling

In this study, we used PARAFAC to fully utilize the fluorescence EEM data of the samples. The PARAFAC model uses alternating least squares to minimize the sum of squares of the residuals of the cubic matrix model to decompose the 3D fluorescence spectroscopy datasets into a set of trilinear terms and an array of residuals [19], as shown in Equation (2):

(2) $$\begin{array}{c} X_{ijk}=\sum_{n=1}^{N}a_{in}b_{jn}c_{kn}+e_{ijk},i=1,\ldots ,I;j=1,\ldots ,J;k=1,\ldots ,K \end{array}$$

where $X_{ijk}$ is an element of a cubic array $X\left(I\times J\times K\right)\;$ with group fraction N, indicating the fluorescence intensity of sample *k* at excitation wavelength i and emission wavelength *j*; N is the number of groups; $a_{in}$, $b_{jn}$, and $c_{kn}$ are the elements of the three basis profile matrices A(I×N), B(J×N), and C(K×N) of X; and $e_{ijk}$ denotes an element of the unfitted three-way residual matrix E of the model.

The PARAFAC modeling was performed using the DOMFluor toolkit in MATLAB R2021b for the parallel factorial modeling analysis of the 3D fluorescence spectral data of the samples.

### 2.5. Machine Learning Models

Random forest (RF) is an algorithm based on the integration of decision trees with Bagging, which introduces random attribute selection during the training process [20,21,22]. For an ordinary decision tree, the algorithm selects one optimal feature among n sample features for region partitioning. In contrast, RF randomly selects a portion of the sample features nsub (nsub < n) on a node and selects one of the optimal features among the randomly selected features to partition the region, repeating the random sampling T times. The final result of RF is an aggregation of the outputs of T weak learners. For the regression algorithm, the regression results obtained from the RF model are the arithmetic mean of the regression results of the T weak learners [23]. The evaluation metrics for type performance include *MSE* and *R*^2^, as shown in Equations (3)–(5):

(3) $$MSE\left(y,\hat{y}\right)=\frac{1}{n}\sum_{i=1}^{n}{\left(y_{i}-{\hat{y}}_{i}\right)}^{2}$$

where $y_{i}$ is the actual value, $\hat{y_{i}}$ is the predicted value, and n is the number of samples in the test set.

(4) $$Var\left(y\right)=\sum_{i=1}^{n}{\left(y_{i}-\hat{y}\right)}^{2}∕n$$

where Var(y) is the variance of the actual value and $\hat{y}$ is the mean of BOD test sample data.

(5) $$R^{2}\left(y,\hat{y}\right)=1-\frac{MSE(\hat{y},y)}{Var(y)}$$

where $R^{2}$ is the coefficient of determination.

### 2.6. Statistical Analysis

The total number of data used for statistical analysis in this study was 240. Statistics were calculated using MATLAB R2021b for parsing the 3D fluorescence spectral data of the water samples (preprocessing, component model identification, split-half test, visualization, etc.). IBM SPSS Statistics 26 and Python 3.7 software were used for the statistical analysis of the data and modeling (non-parametric tests, correlation, random forest model, etc.). Origin Pro 9.1 was used for fitting analysis (linear, exponential, nonlinear) and graphical analysis.

## 3. Results and Discussion

### 3.1. BOD_5_ Analysis of Water Samples

All water samples were tested for BOD at eight-hour intervals and the results are shown in Table S1. The experimental results revealed variations in BOD_5_ values across different sampling points, ranging from a minimum of 3.49 at sampling point 7 to a maximum of 5.42 at sampling point 10. The lowest BOD_5_ value observed at sampling point 7 may be attributed to the faster water flow in the area, fewer pollution sources, or the water body’s enhanced self-purification capacity. In contrast, the highest BOD_5_ value of 5.42 at sampling point 10 likely reflects the influence of nearby pollution sources, such as domestic sewage or areas with high organic matter deposits. These conditions result in significant oxygen consumption by microorganisms decomposing the organic matter. Similarly, sampling points 9 and 11 recorded BOD_5_ values of 5.32 and 5.13, respectively, both in close proximity to sampling point 10, suggesting that this region is likely the most polluted section of the river system. Elevated BOD_5_ values in such areas are often associated with nutrient accumulation, which can stimulate excessive algal growth and potentially lead to algal blooms.

**Figure 2:**
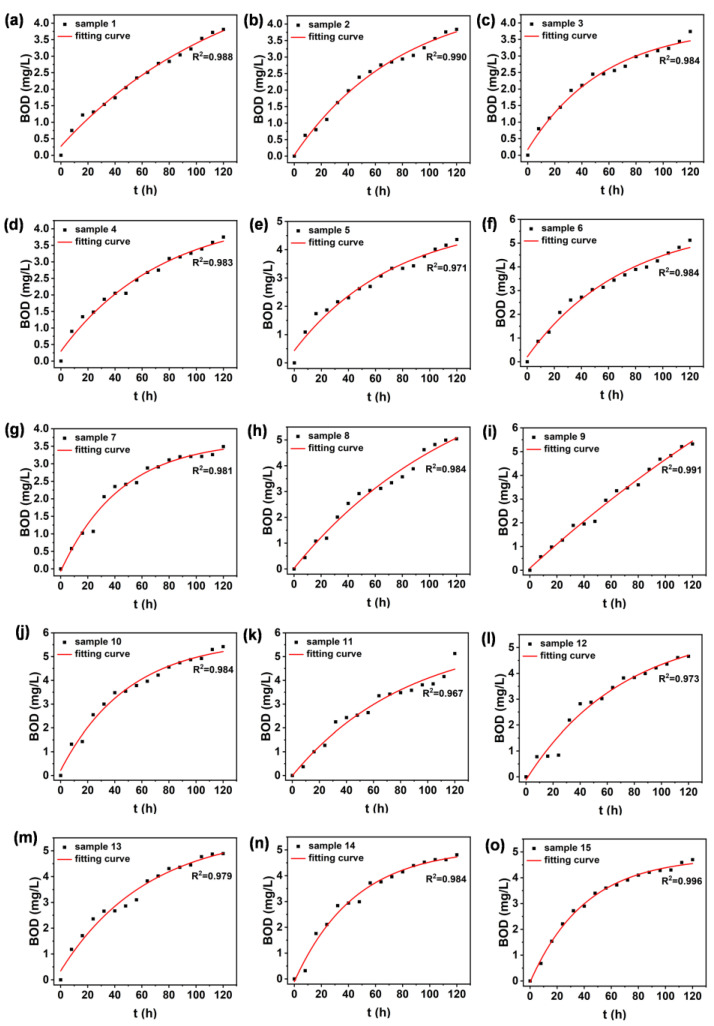
The nonlinear fitting between incubation duration and BOD_t_ of water samples. Panel (**a**–**o**) corresponds to water sample 1–15, respectively.

The samples from all sampling points were fitted exponentially based on the relationship between incubation duration and value, respectively. The results of the fitting are shown in Figure 2. The R^2^ after fitting was greater than 0.967 for all water samples, which implies that the exponential model can effectively describe the trend of BOD over time. During the first 0–2.5 days, the values grow faster and the biochemical reactions are more intense. This is due to the presence of high concentrations of organic matter in the water column and the proliferation of microorganisms in the water column via consumption and decomposition, resulting in a rapid increase in oxygen consumption. As time increases, the rate of increase in oxygen demand slows down until the BOD value stabilizes. This indicates that the organic matter in the water sample is being consumed in a gradual decomposition process and that the microbial activity is close to reaching equilibrium. This phenomenon is consistent with typical BOD growth trends. The R^2^ values of all water samples were relatively close, indicating that the microbial decomposition of organic matter conformed to the exponential decay model and was similar for all sampling points. Their water quality conditions and organic content are relatively similar. The change in the BOD_t_ curve also reflects the self-purification process of the water body. Most of the oxygen-consuming pollutants are attenuated as they migrate or are transformed in the water body, and the water body is thus gradually restored to cleanliness [24].

### 3.2. Fluorescence Spectral Analysis

#### 3.2.1. Three-Dimensional Fluorescence Spectra

**Figure 3:**
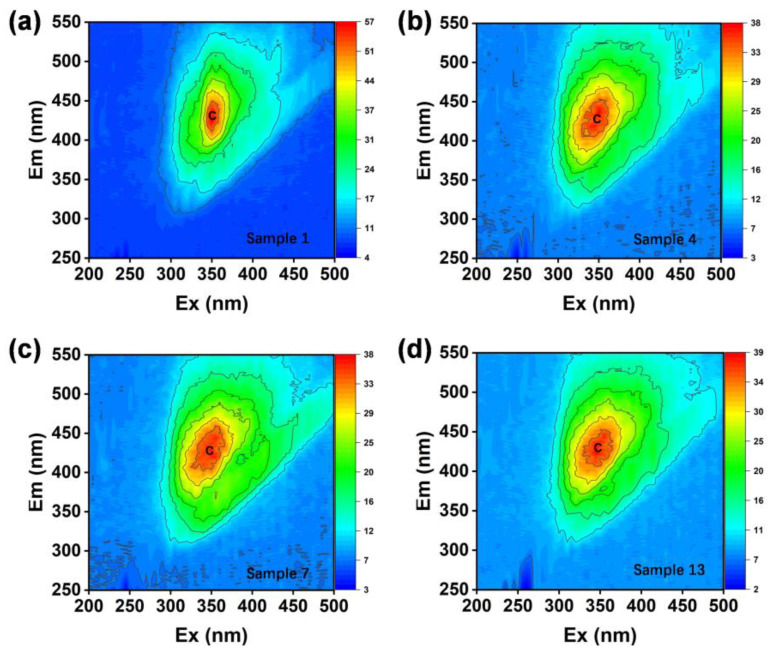
Example of excitation–emission matrix fluorescence spectra of water samples 1, 4, 7, and 13, showing the presence of humic-like DOM peaks (peak C). Panels (**a**–**d**) show the water samples 1, 4, 7, and 13, respectively.

A simple preliminary identification of the general characteristics of the water samples can be achieved with EEMs, using the traditional “peak picking” method, which is intuitive and time-saving [25]. It is necessary to decompose deeper components using PARAFAC analysis. Excitation wavelength (Ex) is the wavelength of light used to excite the fluorescent molecules in the sample, and emission wavelength (Em) is the wavelength of fluorescence emitted by the sample after being excited. As shown in Figure 3, a distinct humic-like fluorophore signal peak (Peak C) appeared in the 3D fluorescence spectrograms of the water samples from the randomly selected sampling points 1, 4, 7, and 13. This represents terrestrial, anthropogenic, or agricultural sources, and is a typical fluorescent signal for soluble organic matter in water bodies [26]. The fluorescence peak intensities of water samples from different sampling points were relatively close to each other, which may indicate that the water bodies at the sampling points were subjected to similar types of pollution or sources of organic matter. Sample 1 showed the highest fluorescence intensity, which may indicate that this point has the highest concentration of organics or is associated with a higher pollution load. The 3D fluorescence spectra of the other water samples were all observed to have the characteristic peak C. See Figure S1 in the Supporting Material.

#### 3.2.2. PARAFAC Results for 3D Fluorescence Spectroscopy

**Figure 4:**
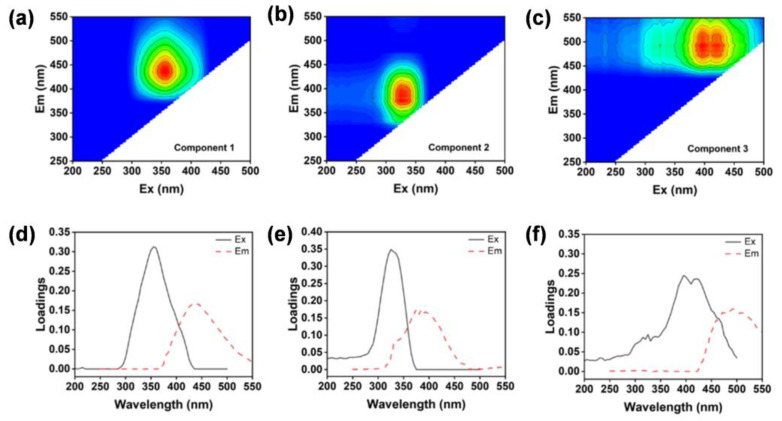
Three components identified using parallel factor modeling. Panel (**a**–**c**) show the fluorescent signatures of components 1–3, respectively; Panel (**d**–**f**) show the excitation and emission spectra of components 1–3, respectively. Different colors represent different spectral intensities, while blue, green, yellow and red colors represent increasing intensities.

The excitation–emission matrix (EEM) fluorescence spectra of all water samples collected were analyzed by parallel factor analysis, and the six samples with higher leverage values were removed. Samples with high leverage have an outsized influence on the PARAFAC model because they deviate significantly from the majority of the data. Removing such samples ensures that the model focuses on the predominant patterns in the dataset. Three macromolecular components were successfully identified from the complete fluorescence data and uploaded to the OpenFluor database to compare the component results. Figure 4 shows the contour plots of the three PARAFAC components.

The EEM spectrum of C1 is characterized by peaks at excitation wavelengths of 350 nm and emission wavelengths of 440 nm, similar to the humic-like fluorescence peak C. It is associated with a group of high-molecular-weight and aromatic molecules of terrestrial origin [26,27,28,29]. The C2 component shows an excitation wavelength maximum of 330 nm and an emission wavelength maximum of 395 nm. Previous studies have linked this peak to marine humus-like substances, which have recently been reported to be related to microbially derived humus-like substances [30,31]. The C3 component, which has a maximum excitation wavelength of 395 and a maximum emission wavelength of 496, may be a fulvic acid of terrestrial origin, but it is not recognizable in conventional peaks [26,32].

### 3.3. Correlation Analysis of Spectral Indicators

**Figure 5:**
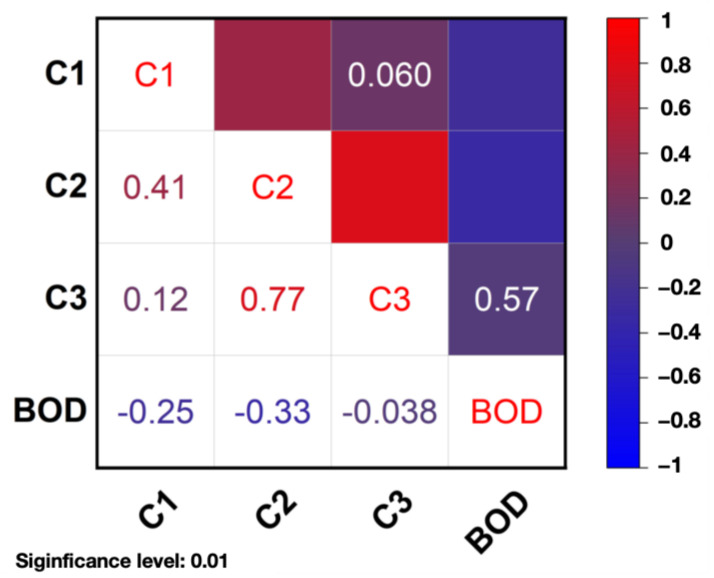
Correlation heatmaps of C1, C2, C3, and BOD.

To compare the correlation coefficients between the maximum fluorescence intensity (Fmax) of the three fractions and BOD_t_ values, Spearman’s value was used in all cases to assess the correlation of each indicator. The correlation thermograms of C1, C2, C3, and BOD are shown in Figure 5. It can be seen that there is a significant negative correlation coefficient between C1 and BOD (R^2^ = −0.20, *p* < 0.01), a significant negative correlation coefficient between C2 and BOD (R^2^ = −0.33, *p* < 0.01), and no significant correlation between the two between C3 and BOD (R^2^ = −0.038, *p* = 0.57). Zhang et al. [33] noted a rapid increase in C2 in phytoplankton degradation experiments. The high positive correlation between C2 and C3 (R^2^ = 0.77, *p* < 0.01) may reflect that they are derived from similar environmental or contamination sources, such as microbial metabolism or co-degradation products of certain organic matter. According to the correlation analysis, C1 and C2 may have some potential to explain the variation in BOD, but the correlation is weak. C3 has little or no correlation with BOD, and further feature selection is required to determine whether to retain C3 when constructing the prediction model.

### 3.4. Construction of Predictive Models

To improve the prediction accuracy in the actual BOD monitoring work, this study integrates all the data measured from 0 to 120 h for 15 water samples. Considering that C3 has no correlation with BODt, further feature selection was used for determination with and without C3. The first group uses t, Fmax(C1), Fmax(C2), and Fmax(C3) as input features and BODt as the output feature, while the second group uses t, Fmax(C1), and Fmax(C2) as input features. The training data were randomly partitioned using the random forest algorithm to merge the two sets of valid data combinations into one large group for training. Among them, 80% are used as training data and 20% as test data, and the training data records the R^2^ and MSE of the test set. The code of python 3.7 for running the Random Forest Vector is shown in the Supplementary Materials.

**Figure 6:**
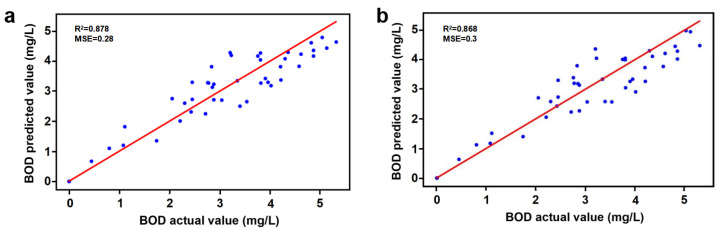
Plot of predicted versus actual values of the random forest regression model: (**a**) with t, C1, C2, and C3 as input feature values; and (**b**) with t, C1, and C2 as input feature values.

Figure 6a,b show the relationship between the predicted and actual values of the random forest model for the first and second sets of data, respectively. The horizontal axis represents the predicted values and the vertical axis represents the actual values. The red diagonal line indicates the ideal fit line, which is the case where the predicted value is the same as the actual value. The scatter points are concentrated around the red diagonal line, indicating that the predicted values of most samples are very close to the actual values. The presence of a few points that slightly deviate from the ideal fit line may be due to measurement noise, prediction bias of the model on some specific samples, or input features that do not fully explain the variation in the target variable BOD.

The results of the random forest model used to predict BOD showed that the model has high goodness of fit and low prediction error. The results of the model in Figure 6a are better predicted than those in Figure 6b because the R^2^ value is larger and the MSE value is smaller. This further illustrates that C3 can be retained as a feature. The R^2^ value of the model with t, C1, C2, and C3 as input features is 0.878, which indicates a relatively strong correlation between the input features (t, C1, C2, C3) and the output feature BODt. The model can fit the relationship between input and output data more accurately. The MSE is 0.28, further indicating that the error between the predicted and actual values is very small. Low MSE values are particularly important because the accurate prediction of BOD in water quality monitoring can provide a reliable basis for management decisions. The model’s more consistent prediction of BOD values in the range of 0 to 5 suggests that it applies to the prediction of water samples with low-to-moderate levels of pollution.

## 4. Conclusions and Future Perspectives

This study proposed a novel method for predicting biochemical oxygen demand (BOD) by integrating 3D fluorescence spectroscopy, parallel factor analysis (PARAFAC), and a random forest (RF) model. BOD values after five days (BOD_5_) and 3D fluorescence spectroscopy data were obtained separately using the Chinese national standard method of incubating water samples. Three distinct components (C1, C2, and C3) were identified from the water samples using PARAFAC. The BOD prediction model was developed using incubation time (t), Fmax (C1), Fmax (C2), and Fmax (C3) as input features, with BOD_5_ values serving as the output variable. The RF model demonstrated high predictive accuracy, achieving a strong goodness of fit (R² = 0.878) and a low mean square error (MSE = 0.28), thereby validating the reliability and accuracy of the proposed method. This approach does not shorten the incubation period required for traditional biochemical oxygen demand assays, but it reduces the reliance on complex experimental steps and provides a convenient, actionable tool for water quality analysis. Combined with the capability of machine learning models, the monitoring system can be further optimized to promote the development of intelligence and automation in water quality testing. However, the applicability of models may be limited by the diversity of environments and the quality of training data, especially in complex or highly polluted water bodies. In the future, it will be necessary to expand its applicability and shorten the detection period by increasing the diversity of training data, improving model accuracy, and incorporating rapid culture techniques. This study provides valuable technical support for efficient water quality monitoring and environmental protection efforts.

In the future, using the whole 3D fluorescence spectra as the input could improve the correlation between the predicted and actual BOD values if the data are effectively preprocessed. However, high-dimensional input data may increase computational cost and the risk of overfitting. This approach warrants further investigation, as it may unlock additional predictive power from the fluorescence data. In addition, while random forests are an excellent starting point due to their robustness and versatility, a comparative analysis with other supervised learning methods could potentially further improve prediction performance. Such an exploration is worthwhile, as it might identify models that achieve a higher correlation between predicted and actual BOD values, thereby improving the utility of the proposed method.

## Figures and Tables

**Table 1:** Common methods for measuring biochemical oxygen demand.

Detection Methods	Principle	Advantages	Disadvantages
National standard method (5-day method)	BOD was calculated by measuring the reduction in dissolved oxygen in water samples after 5 days at 20 °C.	Accurate and reliable, standardized method, internationally recognized, and suitable for a wide range of water quality samples.	Long testing time (5 days), strict requirements on the experimental environment, unable to realize rapid or online testing.
Microbial fuel cell method (MFC)	Electrons generated by microbial metabolism are transferred to the electrodes, generating a current signal that correlates with the BOD value.	Fast detection, short response time, no external power supply required, and suitable for online monitoring.	Microbial cultures require maintenance, sensitivity is affected by temperature and pH, and the detection range is limited.
Electrochemical method	Oxygen electrodes are used to determine the amount of oxygen consumed by microorganisms, or electrochemical sensors are used to detect changes in current or potential associated with BOD values.	Fast response time, portable equipment, easy to operate, and no need for complex sample pre-treatment.	Electrode material selection affects sensitivity and stability, suitable for low BOD concentrations, and complex substrates may interfere with the results.
Biosensor method	Microbial or enzyme-catalyzed metabolic reactions generate detectable signals (current, fluorescence) that correlate with BOD values.	Fast response time, high sensitivity, suitable for low concentration detection, instrument miniaturization, and high portability.	Limited lifetime, requires periodic calibration, and may be damaged by toxic substances in water samples.
Optical method	Indirectly reflecting the biochemical oxygen demand in water samples by monitoring changes in optical signals (fluorescence quenching or absorbance changes, etc.) caused by oxygen consumption during microbial metabolism.	Rapid detection, easy operation, and green environment.	High device cost, low specificity, and limited applicability.

## Data Availability

The data presented in this study are available on request from the corresponding author.

## Supplementary Materials

The following supporting information can be downloaded at https://www.mdpi.com/article/10.3390/s25030711/s1: Python code for random-forest-based machine learning model to predict BOD value. Figure S1: Field photos of 15 sampling sites (a~o). Figure S2: Excitation–emission matrix fluorescence spectra of 15 water samples (a~o) collected at hour 0. Table S1: BOD values for all water samples measured every eight hours for five days.
