# Supplementary material for: Biochemical Oxygen Demand Prediction Based on Three-Dimensional Fluorescence Spectroscopy and Machine Learning
Source: Sensors (Basel). 2025 Jan 24;25(3):711. doi: 10.3390/s25030711 (PMC11821206; doi:10.3390/s25030711)
Supplement: Supplementary file 1 [file sensors-25-00711-s001.zip › sensors-3387508-supplementary.pdf]

Supplementary Materials

Biochemical oxygen demand prediction based on three-dimensional fluorescence spectroscopy and machine learning

Xu Zhang, Yihao Zhang, Xuanyi Yang, Zhiyun Wang* and Xianhua Liu*

School of Environmental Science and Engineering, Tianjin University, Tianjin 300354, PR China

zhangxu_2022@tju.edu.cn (X.Z.); zhangyihao_@tju.edu.cn (Y.Z.); awoelexuan@gmail.com

*Correspondence: zhiyun_wang@tju.edu.cn (Z.W.); lxh@tju.edu.cn (X.L.)

Code and figure captions

Python code for random-forest-based machine learning model to predict BOD value

Figure S1. Field photos of 15 sampling sites (a~o).

Figure S2. Excitation-emission matrix fluorescence spectra of 15 water samples (a~o) collected at hour 0.

Table S1. BOD values for all water samples measured every eight hours for five days.

Python code for random-forest-based machine learning model to predict BOD value

```
import pandas as pd
import numpy as np
from sklearn.ensemble import RandomForestRegressor
from sklearn.model_selection import train_test_split
from sklearn.metrics import mean_squared_error, r2_score
import matplotlib.pyplot as plt
from matplotlib import rcParams
df = pd.read_excel('modeltrainningdata.xlsx')
rcParams['font.sans-serif'] = ['SimHei']
rcParams['axes.unicode_minus'] = False
print(df.head())
features = [ 't','C1', 'C2', 'C3']
X = df[features]
y = df['BOD']
X_train, X_test, y_train, y_test = train_test_split(X, y, test_size=0.2, random_state=42)
model = RandomForestRegressor(n_estimators=100, random_state=42)
model.fit(X_train, y_train)
y_pred = model.predict(X_test)
mse = mean_squared_error(y_test, y_pred)
r2 = r2_score(y_test, y_pred)
print(f"Mean Squared Error (MSE) (MSE): {mse}")
print(f"R²: {r2}")
plt.figure(figsize=(10, 6))
plt.scatter(y_test, y_pred, color='blue', alpha=1)
plt.plot([y_test.min(), y_test.max()], [y_test.min(), y_test.max()], color='red', linewidth=3)
plt.xlabel("actual Value (BOD)mg/L")
plt.ylabel("predicted Value (BOD)mg/L")
plt.title("Random Forest Regression - Predicted Value vs Actual Value")
plt.show()
```

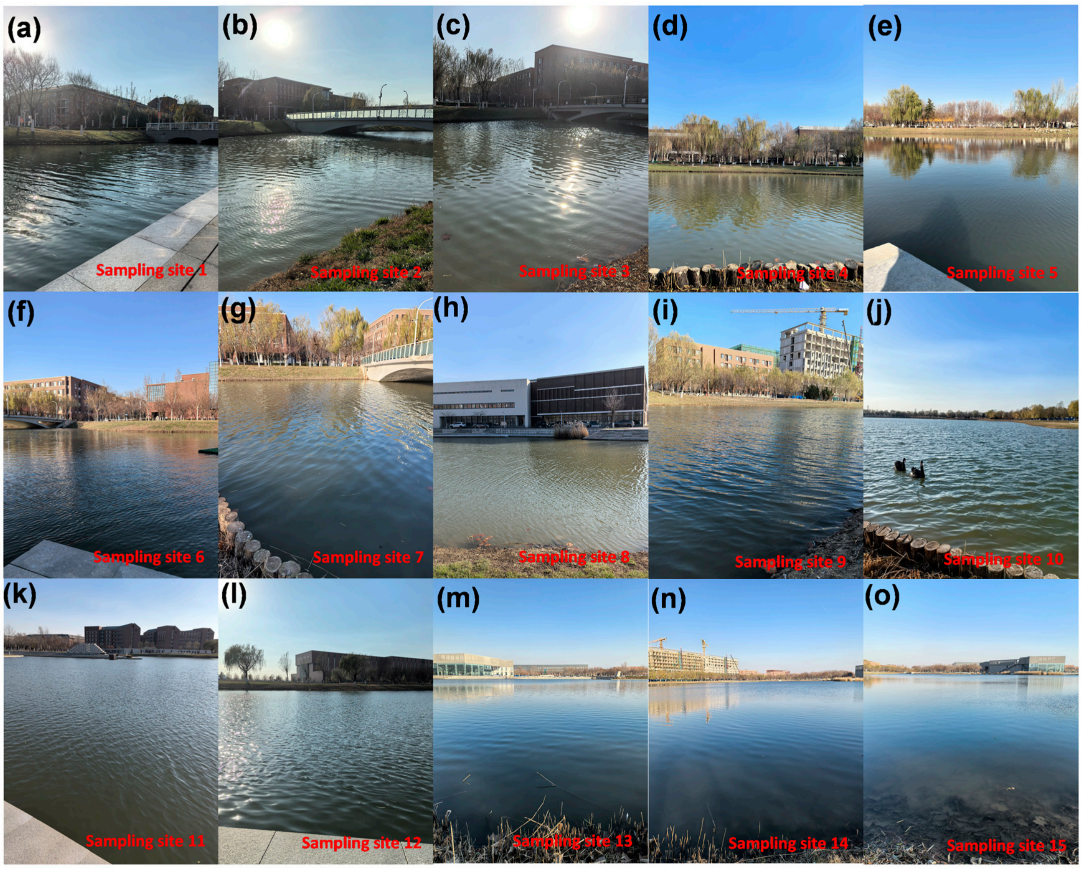

Figure S1. Field photos of 15 sampling sites (a~o).

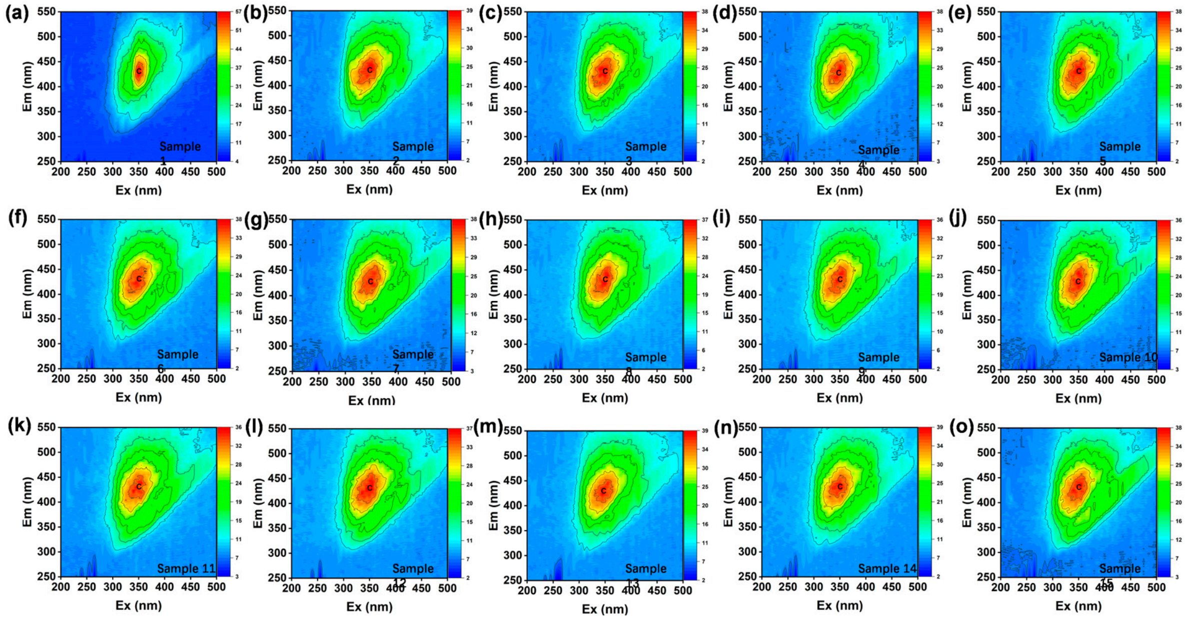

Figure S2. Excitation-emission matrix fluorescence spectra of 15 water samples (a~o) collected at hour 0.

Table S1. BOD values for all water samples measured every eight hours for five days.

water sample	0h	8h	16h	24h	32h	40h	48h	56h	64h	72h	80h	88h	96h	104h	112h	120h
**1**	0	0.75	1.22	1.31	1.54	1.74	2.05	2.34	2.51	2.78	2.84	3.04	3.22	3.54	3.72	3.81
**2**	0	0.63	0.8	1.11	1.62	1.98	2.39	2.56	2.76	2.85	2.94	3.05	3.28	3.56	3.76	3.84
**3**	0	0.8	1.12	1.45	1.96	2.11	2.45	2.46	2.56	2.69	2.98	3.01	3.16	3.23	3.44	3.74
**4**	0	0.9	1.34	1.48	1.87	2.05	2.05	2.45	2.68	2.75	3.1	3.15	3.26	3.39	3.59	3.75
**5**	0	1.09	1.74	1.87	2.16	2.3	2.62	2.7	3.07	3.34	3.34	3.43	3.77	4.02	4.16	4.36
**6**	0	0.86	1.25	2.08	2.6	2.72	3.04	3.14	3.44	3.66	3.89	3.99	4.25	4.58	4.82	5.12
**7**	0	0.58	1.02	1.07	2.06	2.35	2.41	2.46	2.88	2.91	3.11	3.2	3.21	3.21	3.26	3.49
**8**	0	0.44	1.08	1.19	2.01	2.54	2.92	3.04	3.12	3.34	3.57	3.88	4.62	4.82	4.99	5.04
**9**	0	0.57	0.98	1.27	1.89	1.95	2.06	2.95	3.35	3.47	3.6	4.25	4.68	4.83	5.21	5.32
**10**	0	1.32	1.43	2.55	3	3.48	3.54	3.78	3.96	4.22	4.56	4.74	4.87	4.92	5.3	5.42
**11**	0	0.37	1	1.27	2.25	2.43	2.53	2.64	3.35	3.42	3.48	3.58	3.81	3.85	4.16	5.13
**12**	0	0.78	0.8	0.84	2.19	2.82	2.88	3.02	3.45	3.82	3.84	3.99	4.21	4.36	4.61	4.66
**13**	0	1.18	1.71	2.36	2.66	2.67	2.86	3.1	3.83	4.02	4.31	4.36	4.45	4.77	4.87	4.89
**14**	0	0.32	1.76	2.11	2.84	2.94	2.99	3.72	3.76	3.96	4.15	4.39	4.52	4.62	4.62	4.81
**15**	0	0.68	1.54	2.21	2.72	2.9	3.4	3.6	3.72	3.91	4.1	4.21	4.28	4.3	4.59	4.7
